# Transcriptional Regulation in the Immune System: One Cell at a Time

**DOI:** 10.3389/fimmu.2019.01355

**Published:** 2019-06-14

**Authors:** Ananda L. Roy

**Affiliations:** National Institutes of Health, Laboratory of Molecular Biology and Immunology, Biomedical Research Center, National Institute on Aging (NIH), Baltimore, MD, United States

**Keywords:** transcriptional regulation, gene expression, B cells, single cell analysis, immunity

## Abstract

Transcriptional regulation of cells in the immune system must be strictly controlled at multiple levels to ensure that a proper immune response is elicited only when required. Analysis in bulk, or ensemble of cells, provides a wealth of important information leading to a better understanding of the various molecular steps and mechanisms involved in regulating gene expression in immune cells. However, given the substantial heterogeneity of these cells, it is imperative now to decipher these mechanisms at a single cell level. Here I bring together several recent examples to review our understanding of transcriptional regulation of the immune system via single cell analysis and to further illustrate the immense power of such analyses to interrogate immune cell heterogeneity.

## Introduction

Given that transcriptional regulation plays a critical role in mounting a proper immune response, control of gene expression in various cells of the immune (both adaptive and innate) system at transcriptional level has been studied for decades, which has provided important and fundamental information regarding various control mechanisms as well as identified crucial factors that are necessary for transcriptionally regulating the expression of genes required for mounting appropriate immune responses (1–4). Combining these *ex vivo* studies with *in vivo* studies, primarily through murine models, enabled us to decipher in exquisite details, both molecular mechanisms and physiological steps involved in transducing immune signals to elicit correct immune responses at the right time (1–4). Collectively, these studies have provided insights into the logic that dictates how the adaptive and innate arms of the immune system differ with respect to regulating specific genes at the level of structural and functional folding of the chromatin domains, epigenetic regulations, long-range interactions that bring promoter regions and regulatory enhancers in proximity, specific transcription factors that are necessary for lineage commitment and differentiation, and non-coding RNAs that play pivotal roles in immunity (5, 6). However, while the reductionist approaches of studying regulation of individual genes and gene clusters in a given cell were necessary, they were insufficient because such mechanisms in isolated and/or cultured cells could not lead to a systems level view of gene regulation. The advent of next generation sequencing allowed probing global regulatory processes and genome-wide changes in gene expression during immune responses simultaneously in multiple cell types.

In animal tissue, neighboring cells that are apparently identical turn out to exhibit important differences when significant depth of analysis was achieved via single cell techniques. Originally, single cell techniques were applied in situations where biological sample was limiting. But now, given the high throughput technologies that are at our disposal, profiling hundreds of thousands of heterogeneous cells within a population is possible with relative ease (5, 6). With all these remarkable technological advances in studying cellular heterogeneity and discovering rare cell populations via single cell analysis in animal tissues/organs, the question might still be asked whether we really need to understand human biology at single cell resolution. After all, the human body has been defined over centuries by anatomical landmarks, tissue and organ distributions. The answer might lie in the fact that the bewildering cellular heterogeneity in humans often dictates the diseased states and their origins and subsequent treatment. For instance, two apparently “identical” cells in the same organ might behave differently to therapeutic intervention depending on their molecular and functional states. Hence, a “shotgun” approach to treat all neighboring cells in a given tissue might not be necessary or achieve the precision that we strive to attain in modern medicine. Given these considerations, it is no wonder that the precise anatomical landmarks are insufficient and that molecular and positional information of tissue and organ-resident cells must be understood in greater depth to define the human body and its associated maladies (7).

Despite significant technological advances, our understanding of the gene regulation in the immune system still remains incomplete because there is substantial heterogeneity in the cells constituting the system. Immune cells are diverse with respect to developmental stages, function and cell types (e.g., adaptive vs. innate immune cells) as well as location (e.g., primary vs. secondary lymphoid organs) in addition to circulating immune cells through peripheral blood and lymphatic systems (5, 6). Moreover, the function of primary immune cells, apparently of the same lineage, also frequently depends on their interactions with the secondary non-immune cell types and tissues. An added layer of complexity for specific identification of immune cells is introduced by their clonality: they express signature surface immune receptors with distinct genetic diversity that might functionally respond differently to a distinct set of ligands (6). Due to these complexities and the fact that apparently identical immune cells can function at different locations in the body depending on the nature of the requisite immune response, it is imperative that they be profiled at high resolution to determine if indeed they arise from the same origin and consequently might respond similarly during an immune response (6). Here I outline a few recent studies to illustrate the lessons learned from single cell approaches in immune cells and how they often fill gaps of our understanding of the immune system gathered from ensemble and organismal level analysis. Because single cell analysis is still largely limited to transcriptomic analyses (e.g., Single cell RNA-seq, scRNA-seq), these studies illustrate the immense power but also limitations of such analyses. scRNA-seq has been used to identify and classify cell types. Furthermore, it has also been used to characterize rare cell types and analyze variation of gene expression across distinct cell populations based on their steady state RNA levels. However, the dynamics of precise cellular states that are often transient in nature are more difficult to assess simply based on transcriptomic studies (8). But recent developments in imaging (e.g., single molecule Fluorescent *in situ* Hybridization, FISH), proteomics (with CyTOF and MIBI-TOF) and genomics (e.g., LIANTI) provide substantial hope that these additional methods reporting functional states of a cell would complement RNA-seq to identify novel cell types, and further analyze and assign function to these cells and tissues to perhaps reveal unchartered but promising therapeutic avenues (reviewed in 7).

## Solving Longstanding Immunological Problems Via Single Cell Analysis

Memory B cells of the adaptive immune system are generated following pathogenic infection or vaccination so that they can respond against future infections (9). However, select pathogens find ways to either totally evade or, at a minimum, suppress the adaptive immune response. These conditions often lead to induction of memory B cells that are ineffective in differentiating into antibody secreting plasma cells (9). Given that memory B cells play a critical role in vaccination, understanding the basis for such heterogeneity and identifying pathogen-specific memory B cell repertoire could important clues to improve vaccine development. Single cell transcriptomic (scRNA-seq) analysis and specific gene expression programs associated with such diverse population of memory B cells are providing important information for improving future vaccine development and antibody designing for therapeutic usage (10). Another long-standing problem in human immunity is the precise role of Immunoglobulin E (IgE) antibodies. Although secreted IgE protects against infection, it might also cause major health problems, particularly during allergic reactions (11). Despite the fundamental importance of IgE in health and disease, molecular and structural insights into IgE antibodies remain incomplete. Using single cell RNA-seq, Croote et al. determined the gene expression profiles and alternative splicing patterns of IgE secreting B cells from patients with food allergies (11). Remarkably, these specific transcriptomic signatures and splicing profiles specifically associated with IgE producing B cells exhibited identical patterns in individuals who are unrelated (11). These results suggest that these antibodies or derivatives could be employed as therapeutic agents (11). Furthermore, these results might also lead to further understanding of biochemical roles of IgE antibodies in allergic reactions (11). A recent fascinating study identified heterogeneity in uncommitted hematopoietic progenitors with mixed lymphoid and myeloid potentials by single cell RNA-seq (12). Although such heterogeneity has been known for some time, this study concludes that the decision of lymphoid and myeloid lineage choice surprisingly occurs before the hematopoietic progenitor stages with combined lymphoid-myeloid potential called the early progenitor with lymphoid myeloid potential (EPLM) (12). Furthermore, the apparent multipotency of uncommitted progenitors is due to the presence of four subpopulations within these cells, each with their own developmental potentials that are not necessarily restricted to bipotency for lymphoid and myeloid lineages only (12). These results further underscore the power of single cell transcriptomics in resolving both cellular heterogeneity of immune cells as well as establishing molecular relationships amongst distinct hematopoietic precursors via identifying specific transcriptional signatures associated with them (12).

## Function of Immune Cells in Distinct Organs

As noted in the introduction, immune cells are found at multiple organs/tissues within the human body, raising the question of whether they behave in the same fashion at these discrete locations. Single cell analysis is making it possible to interrogate the identity and infer the function of immune cells found in disparate locations. For instance, although it has been known that immune cells are found in liver, the immunobiology of liver remains poorly understood. A new study mapped the cellular landscape of human liver using tissue dissociation techniques combined with functional assays and scRNA-seq (13). They identified 20 discrete types of hepatocytes and other cell populations including B, T, monocyte/macrophage and NK cell types (13). Combining scRNA-seq with image-based approaches provided a detailed spatial map of immune microenvironment of the human liver (13). However, there are several notes of caution raised by the authors. First, these studies show that transcriptional profiling of hepatic cell populations significantly depends on how the liver tissues are prepared as well as the viability of bulk liver homogenate (13). Second, cells dissociated from tissues might behave differently than bulk tissues—in particular, hepatocyte populations are susceptible to dissociation because of the significant heterogeneity of the liver cells (13). Thus, one should take into consideration that not all cell types will be captured with equal efficiency during scRNA-seq analysis (13). Although these single cell mapping efforts identify distinct populations of cells, they do not necessarily identify the “actual frequency of their existence” within the liver tissue of experimental origin (13). Finally, a fact that the community has grappled with in analyzing dissociated cells from tissues is how to define a “normal” tissue. For example, despite the fact that these human liver samples were obtained from “clinically acceptable, healthy liver grafts,” they exhibited mildly inflamed conditions (13). Regardless of these cautionary notes, it is clear that scRNA-seq, combined with other imaging and functional (multi-modal) studies, has immense potential to create detailed cellular maps of human tissues.

Another recent study maps tissue resident macrophages in murine lung and identifies two subpopulations of interstitial macrophages via single cell transcriptomics (14). This study showed that two populations of macrophages in the murine lung were phenotypically distinct and exhibited differences in their intratissue localization (14). While one population of these cells lies close to tissue nerve bundles, the other population is more closely associated with blood vessels and thus, presumed to play a critical role in maintaining blood vessel integrity and antifibrotic activity (14). Although known for some time, these observations show that the immune cells of the same lineage but residing in different locations can function differently depending on the local tissue microenvironment (14). Thus, combining single cell transcriptomics with functional studies and spatial information could lead to identifying novel immune cell populations with characteristic molecular profiles and distinct tissue localizations, presumably performing distinct functions at these locations.

## Identification of Novel Cell Types and States

It is well-known that the vertebrate immune system consists of the innate and adaptive arms, responding to immediate challenge and responding to threats via acquired antigen receptors, respectively. While the innate immune arm in mammals are formed from cells of the myeloid lineage (granulocytes, mast cells, monocytes/macrophages, and dendritic cells), the cells constituting the adaptive immune system are primarily composed of B and T lymphocytes (15). However, the recent discovery of innate lymphoid cells (ILCs) that constitute a rare sub-population of lymphocytes, has challenged this binary notion. Unlike T and B cells, ILCs do not express specific cell surface antigen receptors or undergo clonal expansion when stimulated *ex vivo* (16, 17). Instead, ILCs express cytokine receptors likely to sense environmental threats and rapidly produce a distinct set of cytokines in response to these signals (16–18). Discovery of ILCs has accelerated the need for unbiased methodologies to profile immune cell types solely on the basis of cellular and/or molecular signatures rather than on cell surface markers, because so far immune cells have been traditionally profiled based on surface receptor expression (18). In trying to characterize the immune repertoire in zebrafish, a recent study generated a comprehensive atlas of cellular signatures of lymphocytes defined by their unique transcriptomic profiles in steady state and after challenging the immune system to induce short term inflammation (18). This scRNA-seq analysis led to the surprising finding that zebrafish possesses cytokine producing ILC-like cells much like mammals, potentially involved in responding to environmental threats (18). Thus, scRNA-seq allows to identify heterogeneous cell population and different cellular states in an unbiased fashion based on specific transcriptomic signatures, rather than their surface receptor expression profile (18).

## Single Cell Analysis and Heterogeneity

Heterogeneity in gene expression is important to elucidate because it might indicate the existence of new and yet unidentified subpopulations in such milieu. But, heterogeneity in gene expression profile could also provide novel insights into the function of a given gene or sets of genes (8, 19). For instance, even in an apparent homogeneous population, variation in gene expression will likely arise from stochastic gene expression in addition to various dynamic cellular states like the cell cycle or circadian rhythm (19–21). The steady state level of RNA expression could indicate a static cellular state, but it does not directly reveal status of dynamic processes such as cellular differentiation, cell cycle or circadian rhythm (8, 19–21). In dealing with heterogeneity, one should also be careful about dealing with variability in biological samples, which could be confounding and problematic for further downstream analysis (7, 22, 23). There are two broad types of variability or noise in these experiments: technical variability and biological variability. Technical variability/noise is usually due to changes in sample preparation or processing that might vary depending on the protocols used and experimental conditions (7, 22). In contrast, biological variability might arise due to differences in environmental perturbation or inherent genetic variances of derived biological samples (7, 22).

Additionally, the heterogeneity exhibited in scRNA-seq data could also be due to variability in the expression of a given gene in various cells. This in turn could depend on relative expression state of a given gene compared to other genes in the same signaling pathway (8, 19–21). Variability in level of expression of a given gene across different cells might also reflect how tightly the transcription of this gene is regulated (19). It is now generally believed that nuclear transcription occurs due to the result of RNA polymerase II activity in short bursts giving rise to a set of transcripts, which are processed and transported from the nucleus to the cell cytoplasm for functional usage (24). It then stands to reason that genes resulting from higher transcriptional bursts but lower frequency of expression produce more noise than genes that are expressed due to less frequent transcriptional bursts (19, 24–26). Additionally, it is also shown by Padovan-Merhar et al. that increasing cellular volume or content can result in enhanced transcription because both transcriptional burst size and frequency changes with cellular content/volume and with cell cycle (25). In this regard, it is worth considering an early study of scRNA-seq analysis of bone marrow-derived dendritic cells treated with LPS, which demonstrated extensive bimodal pattern of gene expression and splicing with two distinct patterns of cellular states (27). While the variation is likely due to a number of factors, including developmental stages of the cells, cytokine signaling of a subset of fast-responding bone marrow derived dendritic cells could affect the whole population in part due to changes in transcriptional bursting and alternate splicing (19, 27). In a more recent study, Wu et al. addressed how immunoglobulin (Ig) class switching is triggered in activated B cells (28). Although class switch recombination (CSR) is an important process to generate antibody diversity, the mechanism for transcriptional requirement from upstream promoter region of the Ig constant region (I) for targeting of activation-induced deaminase (AID) enzyme for class switching to IgE and IgG1 remained unclear until recently (28). This study, via single cell analysis in a murine model system, identified an early population of B cells that express Iε but not Iγ1 transcripts in response to IL4 signaling (28). This is likely a result in promoter switching to IgE and occurs at lower levels than Iγ1 (28). Hence, heterogeneity in transcriptional activation of Ig promoters is a likely mechanism responsible for targeting of AID to switch to IgE, which could typify transcriptional activation for many gene networks even in identical and apparently homogenously activated B cells (28).

## Heterogeneity and Alternative Splicing

To understand true transcriptional diversity in cells requires not only to determine total transcript levels but isoform levels as well (5). A computational approach called RNA velocity measures the time derivative of RNA abundance and is capable of distinguishing spliced vs. un-spliced mRNAs in scRNA sequencing analysis. The beauty of RNA velocity is that it can predict the future state of an individual cell on a timescale of hours (29). This appears to be an exciting computational development that could significantly help in analyzing and identifying lineage development and cellular dynamics, which is of particular value when dealing with limited biological samples like human tissues (7, 29). But there are also experimental approaches to identifying splicing variants at single cell level. Using a novel nanopore long-read RNS-seq at single cell level, Byrne et al. experimentally identified thousands of unannotated transcription units, consisting of start and end sites, and hundreds of alternative spliced transcripts in murine B1a cells, suggesting existence of extensive splicing isoforms in these cells (30). Peritoneal cavity derived B-1a cells are distinct from the conventional B2 cells due to their differences in origin of development, their cell surface marker expression and their functions in immune response (31). For example, patients with autoimmune disorders like Systemic Lupus Erythematosus (SLE), Sjogren's syndrome and rheumatoid arthritis exhibit higher levels of B-1 cells when compared to normal subjects (30). Interestingly, hundreds of genes that are specifically expressed in B1a cells exhibit multiple spicing variants, including B cell specific surface receptors, raising the possibility that distinct populations of B1 cells express alternatively spliced protein isoforms, including cell surface receptors, and thus they might respond to different stimuli both quantitatively and/or qualitatively (30). Recognition of such heterogeneity across B1a cell population based on alternative splicing signatures could have important ramifications in better understanding and possible therapeutic potential in treating autoimmune disorders.

## Heterogeneity During Ligand Dependent Differentiation

Immune cells are characterized by surface expression of specific receptors, those that generally respond to signaling via engagement of cognate ligands for differentiation along particular lineages. However, due to high degree of heterogeneity in immune cell lineages, it remains to be determined whether there are sub-populations within a specific lineage that respond differentially (both in a qualitative and quantitative sense) to ligands. To illustrate differential response of immune cells to specific ligands, Chea et al. used single cell analysis, which revealed that there is significant heterogeneity in response of fetal liver-derived ILC progenitors to Notch signaling (32). It is well-known that Notch signaling is required for T cell development, although it is not required for development of fetal liver-derived ILCs (32). Using scRNA-seq, this current work identified two distinct subpopulation of fetal liver-derived ILCs—one that is sensitive to Notch signaling for their proliferation while the other is independent of Notch (32). Hence, the heterogeneity exhibited during ILC development is defined by distinct transcriptional signatures and their differential requirement for Notch signaling (32). Another example of ligand dependent lineage commitment in lymphoid cells was provided by an elegant study by Berthault et al. (33). Given there are multiple distinct stages of differentiation associated with commitment of hematopoietic precursors to lymphoid lineage, the identification of molecular steps involved in this process has been difficult to precisely elucidate (33). Beginning with fetal liver derived precursor cells, this study employed scRNA- seq to elucidate how these cells commit to particular lineage choices by identifying transcriptomic signatures characteristic of B and T cell subsets (33). In particular, identifying the “loss of B cell potential,” which indicate a “T cell bias signature” or a “loss of T cell potential,” indicating a “B cell bias signature” was helpful in characterizing sequential events in this process (33). Surprisingly, majority of precursor cells express both signatures albeit at low levels and such co-expressed signatures persisted through multiple stages of differentiation (33). However, interleukin 7 (IL-7) signaling resolved these lineage choice pathways by quantitatively regulating the lymphoid progenitors via stabilizing the B cell specific transcripts, suggesting a crucial role for cytokine signaling in lymphoid cell fate decisions (33).

## Single Cell Analysis in Aiding Disease Heterogeneity

We now know that many patients do not respond to treatments because recent data shows that roughly 90% of drugs are only effective for < 50% of patients (34). The ineffectiveness could be due to the fact that there is substantial cellular heterogeneity (both across intra- and inter-sample variations) in patient population, which can significantly impact therapy response across multiple cell types and thousands of specific genes (34). Moreover, in contrast to ensemble analysis, single cell analysis could lead to identification of individual clones and associated biomarkers, thereby leading to more precise targeting of each clone (34, 35). For instance, scRNA-seq profiling led to the identification of particular B-cell receptor signaling pathways and gene expression patterns in non-Hodgkin lymphomas (36). Such distinct molecular profiles could possibly explain the differences in therapy response to BCR-pathway inhibitors (36). Likewise, profiling of circulating tumor cells in multiple myeloma via single cell analysis led to further classification of this disease and identification of relevant genes and quantitative assessment of their expression patterns that could be important for future treatment and prognosis (37). It is known that most adult B cell lymphomas exhibit a germinal center B cell phenotype (38). But it remains unclear whether these lymphoma derived B cells retain the functional characteristics of true germinal center B cells or they are halted at certain stages of the germinal center maturation reaction, a notion proposed based on ensemble analysis, which shows a co-expression pattern of follicular- and germinal center B cell-specific genes (38, 39). However, by combining scRNA-seq, phenotypic and genetic analyses of follicular and germinal center-derived B cells with modeling, these studies revealed that although bulk patient samples exhibited mixed profiles of gene expression, germinal center-derived and follicular lymphoma-derived B cells showed distinct transcriptional signatures at higher resolution (39). Hence, they conclude that the B cell lymphoma arises not due to a blockade in a specific stage of germinal center B cell maturation process, but rather these cells have undergone germinal center maturation and acquired novel and dynamic gene expression profiles to increase lymphoma heterogeneity (39).

## Mechanistic Insights From Single Cell Analysis

While single cell assays have been primarily used for identification of heterogeneous or rare cell types, it has not been widely used to determine transcriptional mechanisms. However, to move beyond these important but often descriptive features, single cell analysis must be able to provide significant insights into mechanistic pathways. Indeed, there are some examples of elegantly using single cell analysis to address transcriptional mechanisms. Mostly by combining various *in vitro* assays with transcriptomics and functional assays, these studies demonstrate that the field of single cell analysis could move beyond descriptive analysis to providing mechanistic insights. Rothenberg and colleagues used a combination of scRNA-seq, *in vitro* differentiation assays along with flow cytometry and time-lapse live cell imaging to address lineage commitment mechanisms during T cell development (40). The transcription factor Bcl11b is expressed in all T cell lineages and necessary for commitment to such lineages from precursor cells but the mechanism of how this factor is turned on and maintain expression throughout T cell lineages remained unclear (40). This study identified three distinct steps to turn on *Bcl11b* expression: (i) an early commitment step, where the locus becomes “poised” for expression, which is dependent on two T cell lineage-restricted transcription factors, TCF-1 and GATA-3, (ii) a more “permissive” step that is dependent on Notch signaling, and (iii) a third “amplitude-control” step to modulate *Bcl11b* gene expression, that requires another transcription factor, Runx1, already present from early precursor cells (40). These stepwise and stage-specific mechanisms act in an orchestrated fashion, thereby tightly regulating transcriptional activation of *Bcl11b* that is necessary for developmental commitment of T cell lineage (40). Another study comprehensively characterized transcriptional and differentiation regulation of myeloid progenitor populations *de novo* (41). They show that simply analyzing cell populations by their cell surface receptor expression does not accurately reflect sub-populations of progenitor cells (41). However, by adopting a multi-modal approach, including scRNA-seq, fluorescent activated cell sorting (FACS), functional assays, chromatin profiling (using H3K4me2 as a mark), genetic perturbation, and computational modeling, the authors could profile myeloid cell precursor sub-populations and further suggest that transcriptional priming in myeloid cells is coupled with *in vivo* developmental commitment (41). Their model also proposes a circuitry of potential transcription factor activity within and between myeloid sub-populations (41). Therefore, a combination of genetic perturbation with scRNA-seq and computational modeling enables to further identify the critical players of transcriptional programs during the myeloid differentiation process (41). The same group also used a novel technique called, CRISP-Seq, that combines CRISPR-pooled techniques with scRNA-seq to study the transcriptional pathways regulating bifurcation of monocyte/macrophage and dendritic cell lineages (42). This study identified two critical transcription factor, Cebpb and Irf8 that are critical for such lineage choices and further illustrated the potential of such a highly multiplexed screening strategy to identify “transcriptional rewiring” often associated with inflammatory and antiviral pathways (42).

To elucidate the regulatory check points of B cell development from early hematopoietic precursors through to naïve B cells, Pe'er et al. combined single-cell mass-cytometry together with a computational algorithm to construct developmental trajectories to monitor this progression (43). This comprehensive analysis of human B lymphoid developmental stages allowed them to uncover previously unidentified subsets of B cells that undergo immunoglobulin gene rearrangement by aligning protein co-expression profiles (43). Phenotypically ordering these various stages, they could also identify the role of IL7 mediated phosphorylation of STAT5 in defining these developmental sub-populations of B cells (43). Hence, by combining computational algorithms with scRNA-seq, they identified cellular checkpoints during B cell development that were coordinated with other cellular events like cell cycle status, apoptosis and IgH gene rearrangement, thereby establishing a more complete “ordered” model of B cell development (43). In a more recent study, Miyai et al. used scRNA-seq analysis to unravel mechanism of transcriptional priming of multipotent hematopoietic progenitors to B cell lineage (44). While it is known that stem cell fate is primarily dictated by a set of core transcription factors and associated epigenetic changes, the transcriptional regulatory mechanisms and the cross-talk amongst these transcription factors involved during cell fate decisions remain incomplete (44). A multi-modal approach, which included single cell analysis, demonstrated an unexpected multi-step, sequential transcriptional priming process occurring in three waves, before the regulatory cross-talk begins for B cell commitment (44). The early-wave include activation of transcription factor genes like *Fos* and *Jun*, a mid-wave exhibited upregulation of factors like *Cebpb* and *Tead2* and finally a late-wave that included factors like *SpiB* and *Irf4* as well as genes encoding chromatin regulators like *Ezh2* (44). It is known that scRNA-seq usually suffers from under-representation of lowly expressed mRNAs, which is generally termed “dropout” that hides important relationships amongst various genes and transcriptional pathways in a given cell, thereby limiting accurate mechanistic predictions (45). An algorithm to predict gene interaction pathways and transcription factor targets has been recently developed, which is expected to greatly aid in analyzing scRNA-seq data and accurately deduce even lowly expressed mRNAs in such datasets (45). Taken together, these multi-modal approaches clearly show that in the near future more studies combining scRNA-seq with functional and perturbation experiments as well as computation will be undertaken to move beyond phenomenology and identify transcriptional regulatory mechanisms in the immune system (5).

## Future Perspectives

The bewildering complexity of the mammalian immune system for proper function and appropriate responses to foreign pathogens at the right time is regulated by an elaborate network of cellular and tissue interactions. Hence, to achieve a systems level understanding and mechanistic elucidation of the immune system necessitates identification and characterization of its resident cells with their substantial heterogeneity. Clearly, single cell analysis, though mostly in the realm of transcriptomics/RNA-seq, is providing us with tools to achieve such a feat (46). In the near future, when the single cell data sets are compared and combined with ensemble level profiles from various patient population (e.g., The Cancer Genome Atlas, TCGA; http://cancergenome.nih.gov/), they are likely to identify molecular targets and novel avenues appropriate for therapeutic interventions (47). However, a limitation of over relying on transcriptomic studies is that it is naturally assumed that the message levels accurately correspond to protein levels (48). This is often not true, and the field of single cell analysis needs to advance beyond transcriptomics to technologically develop and subsequently incorporate single cell proteomics, metabolomics, lipid profiles and imaging at high throughput scale comparable to and compatible with RNA-seq (47, 48). Moreover, the studies of isolated, dissociated cells must be combined with *in situ* single cell studies in tissues and organs to provide more meaningful spatial and cellular residency data.

It should be noted though that single cell analysis has come a long way and recent developments in this space raise considerable hope that these studies will move beyond their current, predominantly discovery-driven realm to a mechanistic and hypothesis-driven realm and the mysteries of the immune system will ultimately be resolved. For example, high throughput single cell chromatin contact analysis (Hi-C) is enabling us to decipher the genome architecture in distinct cell types at single cell resolution, which when combined with transcriptomic and proteomic data should provide mechanistic insights into cellular heterogeneity and differentiation (49). New and exciting development in this field now also provides a pathway to carry out cellular profiling via genome topology as the only variable (50). Because the 3D genome structure is usually of high information content with many molecular features, it could be employed in cluster analysis to profile distinct cellular types (50). For example, the promoter-enhancer looping is known to regulate differential gene expression in a cell type dependent fashion. Employing the feature of differentially formed but established cell type–specific promoter-enhancer loops (based on cell type–purified bulk Hi-C), this study could unambiguously separate the single cells into specific clusters of immune cell types (50). Finally, an exciting new advancement called super resolution chromatin tracing that uses super resolution microscopy is revealing at single cell level how the genome is folded into topologically associated domains (TADs) and cooperative interactions at this level even in the absence of cohesion (a co-factor necessary for binding of CCCTC-binding factor CTCF), thus, TADs are likely to be units of chromatin folding (51). This is a breakthrough in studying structure and function of the genome and expected to significantly advance this field (51).

It is worthwhile to ponder why no two cells in an animal could be identical. Raj and colleagues argue that in general, there are two reasons why any two cells might differ from each other and these might not be mutually exclusive (48). First, the fate of the cell could be a deterministic outcome—cells receiving distinctly different instructions, leading to different outcomes. Second, a stochastic or probabilistic outcome– cells behave functionally differently with distinct outcomes, although they receive the same set of instructions. In case of the immune cells, we could perhaps imagine an additional scenario that depending on whether the immune cells of the same lineage landing in distinct anatomical locations “acquire” new functions depending on the “new” tissue niche (changing from deterministic to stochastic fate) or “inherit” distinct functions (remain deterministic) even before they arrive at their final destination. Certainly, we are into really exciting times!

## Author Contributions

The author confirms being the sole contributor of this work and has approved it for publication.

### Conflict of Interest Statement

The author declares that the research was conducted in the absence of any commercial or financial relationships that could be construed as a potential conflict of interest. The handling editor declared a shared affiliation, though no other collaboration, with the author.

## References

[1] Smale ST Fisher AG. Chromatin structure and gene regulation in the immune system. Annu Rev Immunol. (2002) 20:427–62. 10.1146/annurev.immunol.20.100301.064739 11861609

[2] Amit I Regev A Hacohen N. Strategies to discover regulatory circuits of the mammalian immune system. Nat Rev Immunol. (2011) 11:873–80. 10.1038/nri3109 22094988 PMC3747038

[3] Smale ST Tarakhovsky A Natoli G. Chromatin contributions to the regulation of innate immunity. Annu Rev Immunol. (2014) 32:489–511. 10.1146/annurev-immunol-031210-101303 24555473

[4] Rothenberg EV. Transcriptional control of early T and B cell developmental choices. Annu Rev Immunol. (2014) 32:283–321. 10.1146/annurev-immunol-032712-100024 24471430 PMC3994230

[5] Tanay A Regev A. Scaling single-cell genomics from phenomenology to mechanism. Nature. (2017) 541:331–8. 10.1038/nature21350 28102262 PMC5438464

[6] Stubbington MJT Rozenblatt-Rosen O Regev A Teichmann SA. Single-cell transcriptomics to explore the immune system in health and disease. Science. (2017) 358:58–63. 10.1126/science.aan6828 28983043 PMC5654495

[7] Roy AL Conroy RS. Toward mapping the human body at a cellular resolution. Mol Biol Cell. (2018) 29:1779–85. 10.1091/mbc.E18-04-0260 30058989 PMC6085824

[8] Eberwine J Kim J. Cellular deconstruction: finding meaning in individual cell variation. Trends Cell Biol. (2015) 25:569–78. 10.1016/j.tcb.2015.07.004 26410403 PMC4584424

[9] Weisel F Shlomchik M. Memory B cells of mice and humans. Annu Rev Immunol. (2017) 35:255–84. 10.1146/annurev-immunol-041015-055531 28142324

[10] Shah HB Smith K Wren JD Webb CF Ballard JD Bourn RL. Insights from analysis of human antigen-specific memory B cell repertoires. Front Immunol. (2019) 9:3064. 10.3389/fimmu.2018.03064 30697210 PMC6340933

[11] Croote D Darmanis S Nadeau KC Quake SR. High-affinity allergen-specific human antibodies cloned from single IgE B cell transcriptomes. Science. (2018) 362:1306–9. 10.1126/science.aau2599 30545888

[12] Alberti-Servera L von Muenchow L Tsapogas P Capoferri G Eschbach K Beisel C. Single-cell RNA sequencing reveals developmental heterogeneity among early lymphoid progenitors. EMBO J. (2017) 36:3619–33. 10.15252/embj.201797105 29030486 PMC5730887

[13] MacParland SA Liu JC Ma XZ Innes BT Bartczak AM Gage BK. Singe Cell RNA sequencing of human liver reveals distinct intrahepatic macrophage populations. Nat Commun. (2018) 9:4383. 10.1038/s41467-018-06318-7 30348985 PMC6197289

[14] Chakarov S Lim HY Tan L Lim SY See P Lum J. Two distinct interstitial macrophage populations coexist across tissues in specific subtissular niches. Science. (2019) 363:eaau0964. 10.1126/science.aau0964 30872492

[15] Boehm T. Evolution of vertebrate immunity. Curr Biol. (2012) 22:R722–32. 10.1016/j.cub.2012.07.003 22975003

[16] Artis D Spits H. The biology of innate lymphoid cells. Nature. (2015) 517:293–301. 10.1038/nature14189 25592534

[17] Eberl G Colonna M Di Santo JP McKenzie ANJ. Innate lymphoid cells: A new paradigm in immunology. Science. (2015) 348:aaa6566. 10.1126/science.aaa6566 25999512 PMC5658207

[18] Hernández PP Strzelecka PM Athanasiadis EI Hall D Robalo AF Collins CM. Single-cell transcriptional analysis reveals ILC-like cells in zebrafish. Sci Immunol. (2018) 3:eaau5265. 10.1126/sciimmunol.aau5265 30446505 PMC6258902

[19] Kolodziejczyk AA Kim JK Svensson V Marioni JC Teichmann SA. The technology and biology of single-cell RNA sequencing. Mol Cell. (2015) 58:610–20. 10.1016/j.molcel.2015.04.005 26000846

[20] Shalek AK Satija R Shuga J Trombetta JJ Gennert D Lu D. Single-cell RNA-seq reveals dynamic paracrine control of cellular variation. Nature. (2014) 510:363–9. 10.1038/nature13437 24919153 PMC4193940

[21] Buettner F Natarajan KN Casale FP Proserpio V Scialdone A Theis FJ. Computational analysis of cell-to-cell heterogeneity in single-cell RNA-sequencing data reveals hidden subpopulations of cells. Nat Biotechnol. (2015) 33:155–60. 10.1038/nbt.3102 25599176

[22] Stoeckius M Zheng S Houck-Loomis B Hao S Yeung BZ Mauck WM 3rd. Cell Hashing with barcoded antibodies enables multiplexing and doublet detection for single cell genomics. Genome Biol. (2018) 19:224. 10.1186/s13059-018-1603-1 30567574 PMC6300015

[23] Krutzik PO Nolan GP. Fluorescent cell barcoding in flow cytometry allows high-throughput drug screening and signaling profiling. Nat Methods. (2006) 3:361–8. 10.1038/nmeth872 16628206

[24] Lenstra TL Rodriguez J Chen H Larson DR. Transcription dynamics in living cells. Annu Rev Biophys. (2016) 45:25–47. 10.1146/annurev-biophys-062215-010838 27145880 PMC6300980

[25] Padovan-Merhar O Nair GP Biaesch AG Mayer A Scarfone S Foley SW. Single mammalian cells compensate for differences in cellular volume and DNA copy number through independent global transcriptional mechanisms. Mol Cell. (2015) 58:339–52. 10.1016/j.molcel.2015.03.005 25866248 PMC4402149

[26] Kim JK Marioni JC. Inferring the kinetics of stochastic gene expression from single-cell RNA-sequencing data. Genome Biol. (2013) 14:R7. 10.1186/gb-2013-14-1-r7 23360624 PMC3663116

[27] Shalek AK Satija R Adiconis X Gertner RS Gaublomme JT Raychowdhury R. Single-cell transcriptomics reveals bimodality in expression and splicing in immune cells. Nature. (2013) 498:236–40. 10.1038/nature12172 23685454 PMC3683364

[28] Wu YL Stubbington MJ Daly M Teichmann SA Rada C. Intrinsic transcriptional heterogeneity in B cells controls early class switching to IgE. J Exp Med. (2017) 214:183–96. 10.1084/jem.20161056 27994069 PMC5206502

[29] La Manno G Soldatov R Zeisel A Braun E Hochgerner H Petukhov V. RNA velocity of single cells. Nature. (2018) 560:494–8. 10.1038/s41586-018-0414-6 30089906 PMC6130801

[30] Byrne A Beaudin AE Olsen HE Jain M Cole C Palmer T. Nanopore long-read RNAseq reveals widespread transcriptional variation among the surface receptors of individual B cells. Nat Commun. (2017) 8:16027. 10.1038/ncomms16027 28722025 PMC5524981

[31] Rothenberg EV. Multiple curricula for B cell developmental programming. Immunity. (2016) 45:457–58. 10.1016/j.immuni.2016.09.005 27653594

[32] Chea S Schmutz S Berthault C Perchet T Petit M Burlen-Defranoux O. Single-cell gene expression analyses reveal heterogeneous responsiveness of fetal innate lymphoid progenitors to notch signaling. Cell Rep. (2016) 14:1500–16. 10.1016/j.celrep.2016.01.015 26832410

[33] Berthault C Ramond C Burlen-Defranoux O Soubigou G Chea S Golub R. Asynchronous lineage priming determines commitment to T cell and B cell lineages in fetal liver. Nat Immunol. (2017) 18:1139–49. 10.1038/ni.3820 28825702

[34] Shalek AK Benson M. Single-cell analyses to tailor treatments. Sci Transl Med. (2017) 9:eaan4730. 10.1126/scitranslmed.aan4730 28931656 PMC5645080

[35] Wang L Fan J Francis JM Georghiou G Hergert S Li S. Integrated single-cell genetic and transcriptional analysis suggests novel drivers of chronic lymphocytic leukemia. Genome Res. (2017) 27:1300–11. 10.1101/gr.217331.116 28679620 PMC5538547

[36] Myklebust JH Brody J Kohrt HE Kolstad A Czerwinski DK Wälchli S. Distinct patterns of B-cell receptor signaling in non-Hodgkin lymphomas identified by single-cell profiling. Blood. (2017) 129:759–70. 10.1182/blood-2016-05-718494 28011673 PMC5301824

[37] Lohr JG Kim S Gould J Knoechel B Drier Y Cotton MJ. Genetic interrogation of circulating multiple myeloma cells at single-cell resolution. Sci Transl Med. (2016) 8:363ra147. 10.1126/scitranslmed.aac7037 27807282 PMC5426804

[38] Young RM Staudt LM. Targeting pathological B cell receptor signalling in lymphoid malignancies. Nat Rev Drug Discov. (2013) 12:229–43. 10.1038/nrd3937 23449308 PMC7595252

[39] Milpied P Cervera-Marzal I Mollichella ML Tesson B Brisou G Traverse-Glehen A. Human germinal center transcriptional programs are de-synchronized in B cell lymphoma. Nat Immunol. (2018) 19:1013–24. 10.1038/s41590-018-0181-4 30104629

[40] Kueh HY Yui MA Ng KK Pease SS Zhang JA Damle SS. Asynchronous combinatorial action of four regulatory factors activates Bcl11b for T cell commitment. Nat Immunol. (2016) 17:956–65. 10.1038/ni.3514 27376470 PMC4955789

[41] Paul F Arkin Y Giladi A Jaitin DA Kenigsberg E Keren-Shaul H. Transcriptional heterogeneity and lineage commitment in myeloid progenitors. Cell. (2015) 163:1663–77. 10.1016/j.cell.2015.11.013 26627738

[42] Jaitin DA Weiner A Yofe I Lara-Astiaso D Keren-Shaul H David E. Dissecting Immune Circuits by Linking CRISPR-Pooled Screens with Single-Cell RNA-Seq. Cell. (2016) 167:1883–96. 10.1016/j.cell.2016.11.039 27984734

[43] Bendall SC Davis KL Amir el-AD Tadmor MD Simonds EF Chen TJ. Single-cell trajectory detection uncovers progression and regulatory coordination in human B cell development. Cell. (2014) 157:714–25. 10.1016/j.cell.2014.04.005 24766814 PMC4045247

[44] Miyai T Takano J Endo TA Kawakami E Agata Y Motomura Y. Three-step transcriptional priming that drives the commitment of multipotent progenitors toward B cells. Genes Dev. (2018) 32:112–26. 10.1101/gad.309575.117 29440259 PMC5830925

[45] van Dijk D Sharma R Nainys J Yim K Kathail P Carr AJ. Recovering gene interactions from single-cell data using data diffusion. Cell. (2018) 174:716–729.e27. 10.1016/j.cell.2018.05.061 29961576 PMC6771278

[46] Papalexi E Satija R. Single-cell RNA sequencing to explore immune cell heterogeneity. Nat Rev Immunol. (2018) 18:35–45. 10.1038/nri.2017.76 28787399

[47] Stuart T Satija R. Integrative single-cell analysis. Nat Rev Genet. (2019) 20:257–72 10.1038/s41576-019-0093-7 30696980

[48] Symmons O Raj A. What's luck got to do with it: single cells, multiple fates, and biological nondeterminism. Mol Cell. (2016) 62:788–802. 10.1016/j.molcel.2016.05.023 27259209 PMC4900469

[49] Cao J Cusanovich DA Ramani V Aghamirzaie D Pliner HA Hill AJ. Joint profiling of chromatin accessibility and gene expression in thousands of single cells. Science. (2018) 361:1380–5. 10.1126/science.aau0730 30166440 PMC6571013

[50] Tan L Xing D Chang CH Li H Xie XS. Three-dimensional genome structures of single diploid human cells. Science. (2018) 361:924–8. 10.1126/science.aat5641 30166492 PMC6360088

[51] Bintu B Mateo LJ Su JH Sinnott-Armstrong NA Parker M Kinrot S. Super-resolution chromatin tracing reveals domains and cooperative interactions in single cells. Science. (2018) 362: eaau1783 10.1126/science.aau1783 30361340 PMC6535145

